# Dynamic Changes in the Spatiotemporal Localization of Rab21 in Live RAW264 Cells during Macropinocytosis

**DOI:** 10.1371/journal.pone.0006689

**Published:** 2009-08-20

**Authors:** Youhei Egami, Nobukazu Araki

**Affiliations:** Department of Histology and Cell Biology, School of Medicine, Kagawa University, Miki, Kagawa, Japan; University of Birmingham, United Kingdom

## Abstract

Rab21, a member of the Rab GTPase family, is known to be involved in membrane trafficking, but its implication in macropinocytosis is unclear. We analyzed the spatiotemporal localization of Rab21 in M-CSF-stimulated RAW264 macrophages by the live-cell imaging of fluorescent protein-fused Rab21. It was demonstrated that wild-type Rab21 was transiently associated with macropinosomes. Rab21 was recruited to the macropinosomes after a decrease in PI(4,5)P_2_ and PI(3,4,5)P_3_ levels. Although Rab21 was largely colocalized with Rab5, the recruitment of Rab21 to the macropinosomes lagged a minute behind that of Rab5, and preceded that of Rab7. Then, Rab21 was dissociated from the macropinosomes prior to the accumulation of Lamp1, a late endosomal/lysosomal marker. Our analysis of Rab21 mutants revealed that the GTP-bound mutant, Rab21-Q78L, was recruited to the macropinosomes, similarly to wild-type Rab21. However, the GDP-bound mutant, Rab21-T33N, did not localize on the formed macropinosomes, suggesting that the binding of GTP to Rab21 is required for the proper recruitment of Rab21 onto the macropinosomes. However, neither mutation of Rab21 significantly affected the rate of macropinosome formation. These data indicate that Rab21 is a transient component of early and intermediate stages of macropinocytosis, and probably functions in macropinosome maturation before fusing with lysosomal compartments.

## Introduction

Macropinocytosis is a clathrin-independent endocytosis which accounts for the bulk fluid-phase uptake from the extracellular environment, and is an essential aspect of normal cell function. In macrophages and dendritic cells, which display high levels of constitutive macropinocytosis, macropinocytosis plays a central role in antigen presentation [1]–[5]. In fibroblasts and epithelial cells, macropinocytosis is rarely seen, but markedly induced after stimulation by platelet-derived growth factor (PDGF) and epidermal growth factor (EGF), respectively [4], [6], although the physiological role of macropinocytosis in these cell types is largely unknown. In addition, some pathogenic bacteria and viruses exploit macropinocytosis to invade their host [7]–[10]. Owing to this high important physiopathological relevance of macropinocytosis, the regulatory mechanisms of macropinosome formation and maturation have recently been receiving increasing attention.

Macropinosome formation is initiated by actin-dependent cell-surface membrane ruffling. Then, some membrane ruffles form circular ruffles (macropinocytic cups), and close into macropinosomes, which are large endocytic vacuoles (0.2–5 µm), by fission from the plasma membrane. Usually, newly formed macropinosomes gradually mature and finally merge with lysosomes [11]. Both phosphoinositides and small GTPases, such as Cdc42, Rac, ARF6 and Rab5, are known to regulate actin polymerization and remodeling in membrane ruffling and macropinocytosis [3], [12]–[17]. Rab GTPases are key to membrane trafficking that mediates both maturation and vesicle transport through endocytic pathways, including macropinocytosis [4], [11], [18]. Although the actin-dependent process of macropinosome formation is more similar in appearance and regulation to phagocytosis than clathrin-dependent endocytosis, some of the mechanisms in macropinosome maturation are shared with those of the clathrin-dependent endocytic pathway. Knowledge about macropinocytosis has increased; nevertheless, the complex regulatory molecular components and signaling pathways of macropinocytosis still require detailed investigation.

Small GTPases of the Rab family are key to membrane-trafficking events in eukaryotic cells. To date, more than 60 Rab members have been identified in the human genome [19]. Many of the Rab proteins that are localized on distinct compartments have been reported to coordinate sequential steps of membrane transport [20], [21]. In the receptor-mediated, clathrin-dependent endocytic pathway, Rab5, Rab7 and Rab11 are localized on distinct endosomal compartments, and function as marker proteins for early endosomes, late endosomes and recycling endosomes, respectively [18]–[21].

Rab5 regulates membrane traffic into and between early endosomes in clathrin-dependent endocytosis [18]. Also, it has been shown that Rab5 is involved in circular ruffle and macropinosome formation [12], [22]. Although the role of Rab5 in the endocytic pathway is by far the best studied, those of several other members are not yet fully understood. One such member is Rab21. Rab21 mainly localizes on early endosomes and is implicated in endosome dynamics [23]–[26]. A recent study has shown that Rab21 regulates phagocytosis, which shares many features with macropinocytosis, by interacting with two LIM domain proteins, LimF and ChLim, in *Dictyostelium discoideum* [27]. However, it remains unclear whether or not Rab21 is involved in macropinocytosis.

In this study, we found that Rab21 is associated with macropinosomes in RAW264 macrophages expressing Rab21 fused with green fluorescent protein (GFP) variants. We demonstrated the dynamic changes in the spatiotemporal localization of Rab21 during macropinocytosis by fluorescence live-cell imaging.

## Results

### Rab21 is transiently associated with macropinosomes in macrophages

**Figure 1:**
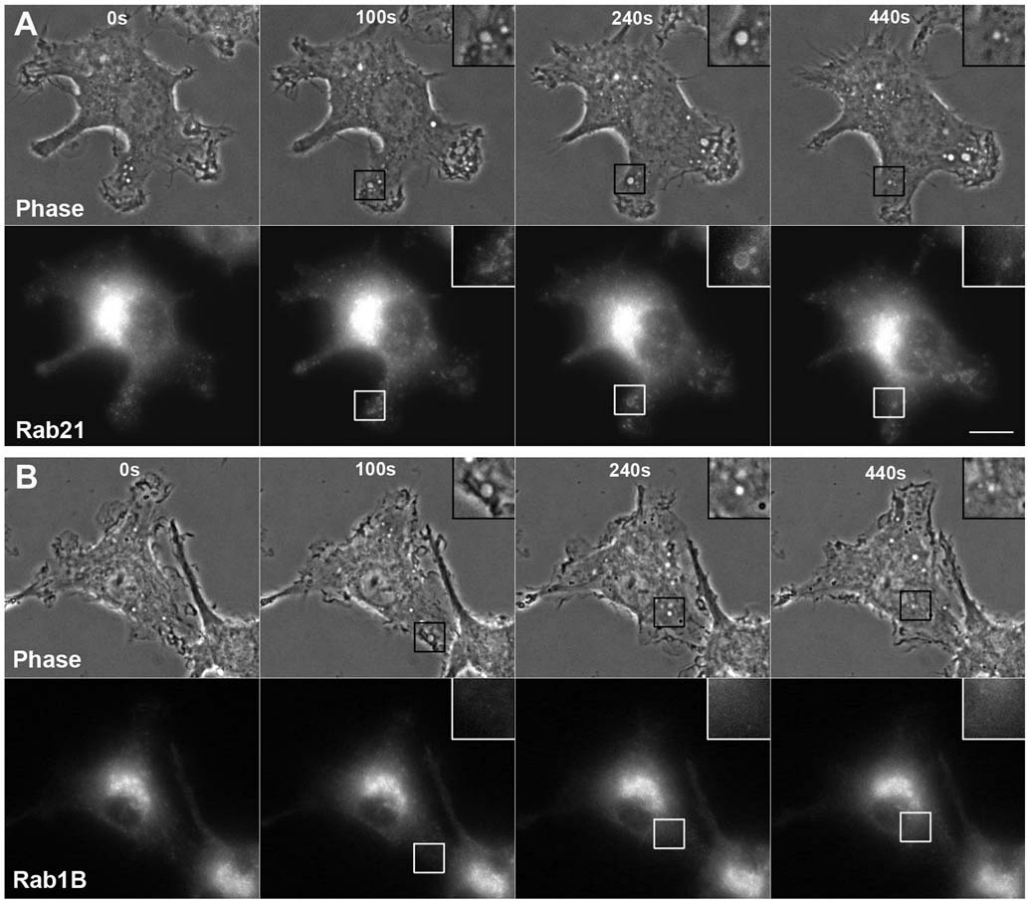
Live-cell imaging of GFP-Rab21 in RAW264 cells during macropinocytosis. (A) Live RAW264 cells expressing GFP-Rab21 after M-CSF treatment were observed by fluorescence microscopy. Time-lapse images were acquired using the MetaMorph imaging system. Phase-contrast images are shown (upper panels). The elapsed time is indicated at the top. The beginning of circular ruffle formation from curved ruffles was set as time 0. It is noteworthy that Rab21 was associated with a formed macropinosome (inset, t = 240 s). Five cells were examined in three independent experiments, and similar results were obtained. The corresponding movie is available (Movie S1). (B) As a control, GFP-Rab1B, a non-endocytic Rab protein, was expressed in RAW264 cells. No association of Rab1B with macropinosomes was observed. Scale bars: 10 µm.

It has been previously demonstrated that Rab5 localizes on macropinosomes [16], [22], [28]. These results prompted us to examine whether or not Rab21, a close homolog of Rab5, also localizes on macropinosomes. We examined the association of Rab21 with macropinosomes in live RAW264 macrophages expressing GFP-Rab21 by phase-contrast and fluorescence microscopy. Time-lapse image analysis showed that the stimulation of macrophages with macrophage colony-stimulating factor (M-CSF) enhanced membrane ruffling and circular ruffle formation. Subsequently, circular cup-shaped membrane ruffles closed into intracellular macropinosomes (Movie S1), as previously reported [29]–[31]. By live-cell fluorescence imaging, GFP-Rab21 was found to be associated with newly formed macropinosomes, but not late macropinosomes older than ∼8 min (Figure 1A and Movie S1). As controls, RAW264 cells were transfected with plasmids expressing GFP alone or GFP-Rab1B, a marker of the ER-Golgi intermediate compartment. Neither GFP nor GFP-Rab1B was recruited to macropinosomes (Figure 1B).

It is known that macrophages constitutively exhibit macropinocytosis under normal conditions, although the macropinocytic activity in unstimulated macrophages was lower than that in M-CSF-stimulated macrophages [29]–[31]. We examined the association of Rab21 with constitutive macropinocytosis in macrophages without M-CSF stimulation. In unstimulated RAW264 cells, it was observed that Rab21 was transiently localized on macropinosomes, similar to that in M-CSF-stimulated cells (Movie S2). These findings indicate that Rab21 is transiently recruited to macropinosomes in macrophages with or without M-CSF stimulation.

### GTP binding of Rab21 is required for its recruitment to macropinosomes but not for macropinosome formation

**Figure 2:**
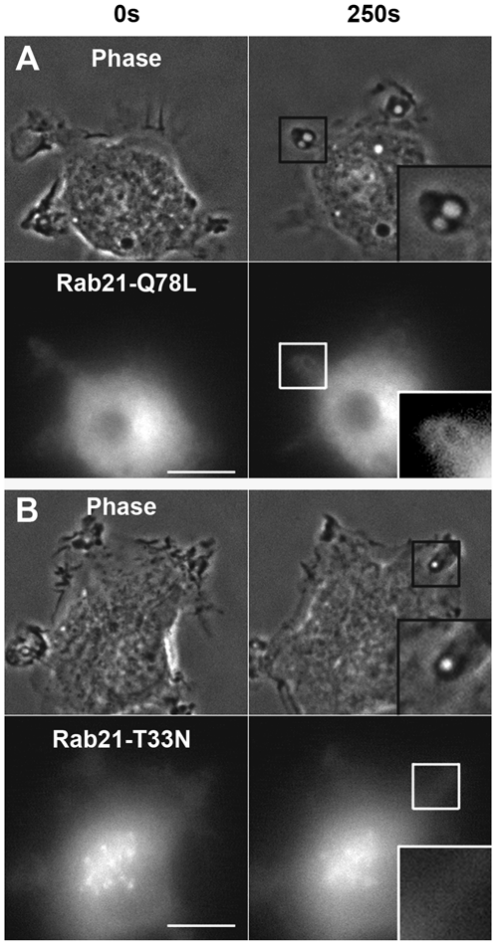
The GDP-bound mutant of Rab21 does not localize on macropinosomes. RAW264 cells were transfected with GFP-Rab21-Q78L, a GTP-bound mutant (A) or GFP-Rab21-T33N, a GDP-bound mutant (B). Time-lapse images were acquired in the same way as for Fig. 1. The elapsed time is indicated at the top. The insets show higher magnification images of the indicated regions of the cells. Images are representative of six cells from two independent experiments. Scale bar: 10 µm.

We examined the intracellular localization of two Rab21 mutants that have an impaired GTP cycle: Rab21-T33N (GDP-bound mutant, defective in GTP binding) and Rab21-Q78L (GTP-bound mutant, defective in GTP hydrolysis). The distribution of GFP-Rab21-T33N in RAW264 cells was diffuse in the cytosol and concentrated in the perinuclear region, as previously shown in other cell types [24]. Even after M-CSF stimulation, no change in the localization of GFP-Rab21-T33N was observed (Figure 2). Although membrane ruffling and macropinosome formation occurred normally, Rab21-T33N was not recruited to the membrane of the macropinosomes. In contrast to GFP-Rab21-T33N, GFP-Rab21-Q78L accumulated on the macropinosomes as prolifically as wild-type Rab21 (Figure 2). These data indicate that GTP-binding is required for the association of Rab21 with macropinosomes.

**Figure 3:**
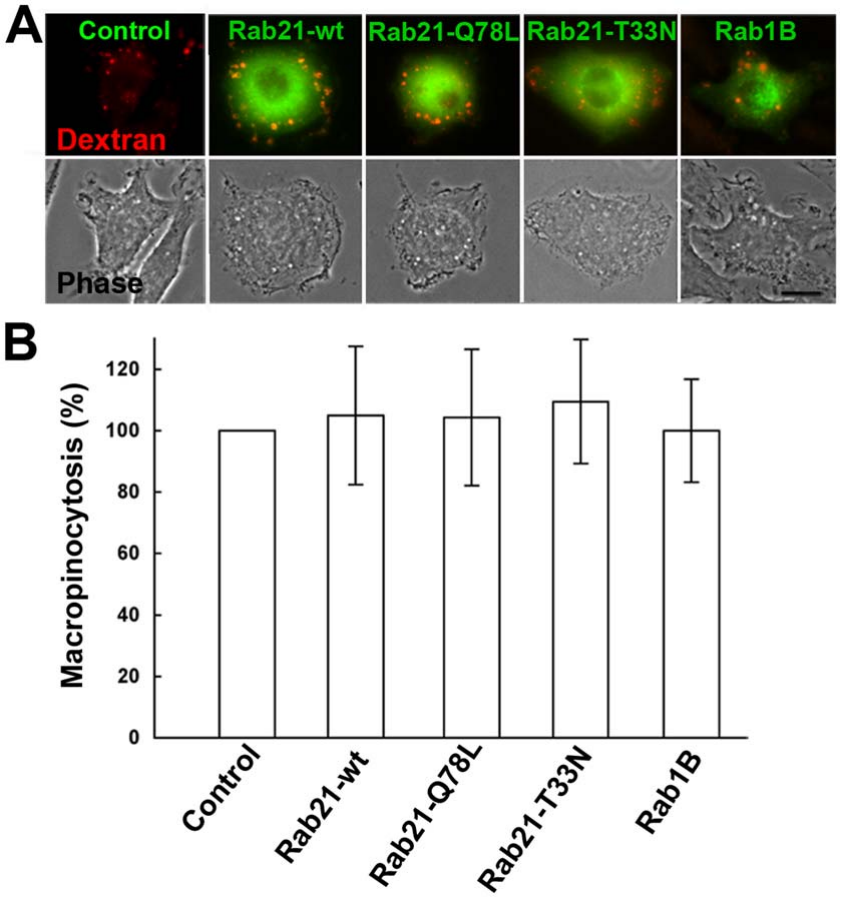
Quantitation of macropinosome formation in RAW264 cells expressing wild-type Rab21, Rab21-Q78L, Rab21-T33N or Rab1B. (A) Macropinocytosis of RITC dextran by RAW264 cells expressing GFP-fused Rab21-wild-type, Rab21-Q78L, Rab21-T33N, or Rab1B were compared with control untransfected cells. The cells were incubated with RITC dextran in the presence of M-CSF for 10 min, and fixed. Images of fluorescence and corresponding phase-contrast are representative of 150 cells from three independent experiments. Red: RITC-dextran; green: GFP-Rab proteins. Scale bar: 10 µm. (B) The number of newly formed macropinosomes per cell (n = 50) was counted under a microscope. Macropinosomes were identified by their size (>1 µm) and content of RITC-dextran, a fluid-phase marker. The values expressed as percentages of the index obtained for control (untransfected cells) are the means±S.E. of triplicate experiments. There was no significant difference in the rate of macropinosome formation between cells expressing either wild-type GFP-Rab21, Rab21-Q78L or Rab21-T33N and the control untranfected cells.

Next, we examined the effect of Rab21 mutation on fluid-phase uptake by macropinocytosis to evaluate the functional implication of its GTP binding and GTP hydrolysis in macropinosome formation. RAW264 cells were incubated with rhodamine B isothiocyanate (RITC) dextran, a fluid-phase marker, in the presence of M-CSF for 10 min. The rate of macropinosome formation was quantified by scoring the number of macropinosomes labeled with RITC-dextran per cell under a fluorescence and phase-contrast microscope. However, quantification of the number of RITC-dextran-labeled macropinosomes per cell revealed no significant change in the number of macropinosomes in the cells expressing GFP-Rab21, Rab21-Q78L or Rab21-T33N as compared to untransfected control cells (Figure 3). Taking these facts into account, we conclude that the binding of GTP to Rab21 is not crucial for macropinosome formation, but is required for the recruitment of Rab21 to the macropinosomes.

### Rab21 is associated with macropinosomes after a decrease in PI(4,5)P_2_ and PI(3,4,5)P_3_ levels

**Figure 4:**
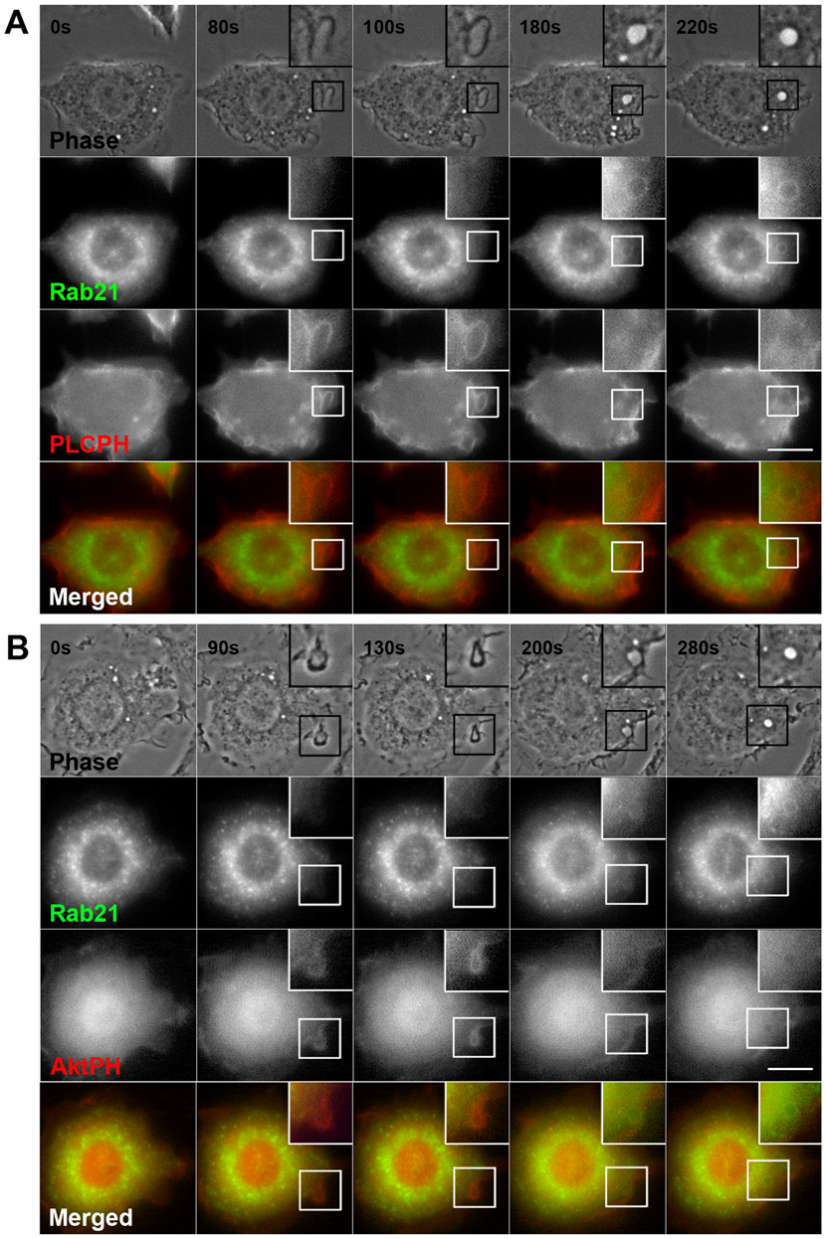
Rab21 is recruited onto macropinosomes after the loss of PI(4,5)P_2_ and PI(3,4,5)P_3_. (A) RAW264 cells were co-expressing GFP-Rab21 (green) and CFP-PLC-PH (red), which shows the localization of PI(4,5)P_2_. Time-lapse images were obtained under the same conditions as for Fig. 1. Phase-contrast images are shown in the top panels. These time-lapse images are representative of three independent experiments. Scale bar: 10 µm. The corresponding movie is available (Movie S3). (B) The time-lapse images of RAW264 cells co-expressing GFP-Rab21 (green) and CFP-Akt-PH (red), which shows the localization of PI(3,4,5)P_3_, were acquired in the same way as for Fig. 3A. The time-lapse images are representative of four independent experiments. Scale bar: 10 µm. The corresponding movie is available (Movie S4).

We have recently investigated the time course of the phosphoinositide metabolism in live A431 cells during macropinocytosis [13]. The phosphatidylinositol 4,5-bisphosphate [PI(4,5)P_2_] levels in the plasma membrane increase immediately after EGF stimulation to induce the formation of ruffles, and decrease at once when the circular ruffles close into macropinosomes. Meanwhile, the phosphatidylinositol 3,4,5-triphosphate [PI(3,4,5)P_3_] levels increase in the membrane at the time of macropinosome closure and decrease after a few minutes. In order to clarify the time of Rab21 recruitment to the macropinosomes, we co-expressed GFP-Rab21 with cyan fluorescent protein (CFP) probes specific for phosphoinositides, such as PI(4,5)P_2_ and PI(3,4,5)P_3_, in RAW264 cells. Observations of live cells co-expressing GFP-Rab21 and the CFP-phospholipase Cδ1 (PLC)-pleckstrin homology domain (PH) which binds to PI(4,5)P_2_ revealed that Rab21 was recruited to the macropinosomes after PI(4,5)P_2_ fell away from the membrane (Figure 4A and Movie S3). Next, we investigated whether or not PI(3,4,5)P_3_ was also released from the macropinosomes before Rab21 recruitment by using GFP-Rab21 and CFP-Akt-PH, which binds PI(3,4,5)P_3_ preferentially. We observed that the loss of PI(3,4,5)P_3_ from the macropinosomal membrane was followed by Rab21 recruitment (Figure 4B and Movie S4). These findings indicate that Rab21 recruitment onto macropinosomes occurs later than the diminution of the PI(4,5)P_2_ and PI(3,4,5)P_3_ levels.

### The association of Rab21 with macropinosomes is slightly preceded by that of Rab5 and followed by that of Rab7

**Figure 5:**
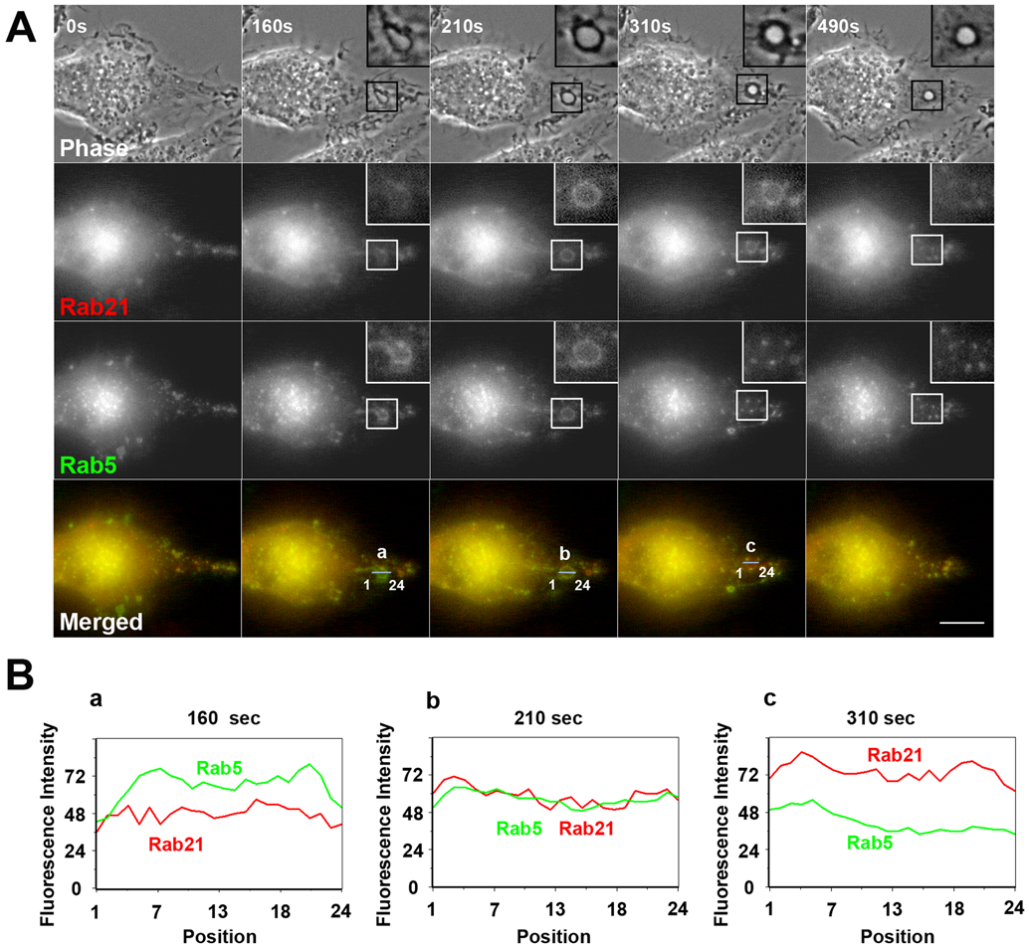
The recruitment of Rab21 to the macropinosomes is preceded by that of Rab5. (A) Live RAW264 cells co-expressing CFP-Rab21 and YFP-Rab5 were observed by digital fluorescence microscopy. The elapsed time is indicated at the top. Representative images from three independent experiments are shown. The insets show higher-magnification images of the indicated regions of the cells. Scale bar: 10 µm. The corresponding movie is available (Movie S5). (B) Line scan analysis of the fluorescence intensities of YFP-Rab5 (green) and CFP-Rab21 (red) through a macropinosome at t = 160 sec (a), t = 210 sec (b), t = 310 sec (c). The beginning of circular ruffle formation from curved ruffles was set as time 0.

It is known that Rab21 shows high homology with endocytic Rab5, as compared to all the other Rab family members [24]. Therefore, we transiently co-transfected RAW264 cells with YFP-Rab5 and CFP-Rab21, and compared the localizations of Rab21 and Rab5 during macropinocytosis. As expected, Rab21 and Rab5 were largely colocalized on macropinosomes in RAW264 cells. However, it was found that the recruitment of Rab21 to the macropinosomes occurred slightly later than that of Rab5 (Figure 5 and Movie S5). Line scan analysis of the fluorescence intensities of YFP-Rab5 and CFP-Rab21 clearly indicated that the peak of association of Rab5 with macropinosomes is earlier than that of Rab21 (Figure 5B). In addition, the association of Rab21 with the macropinosomes persisted even after the loss of Rab5 from the macropinosomal membrane.

**Figure 6:**
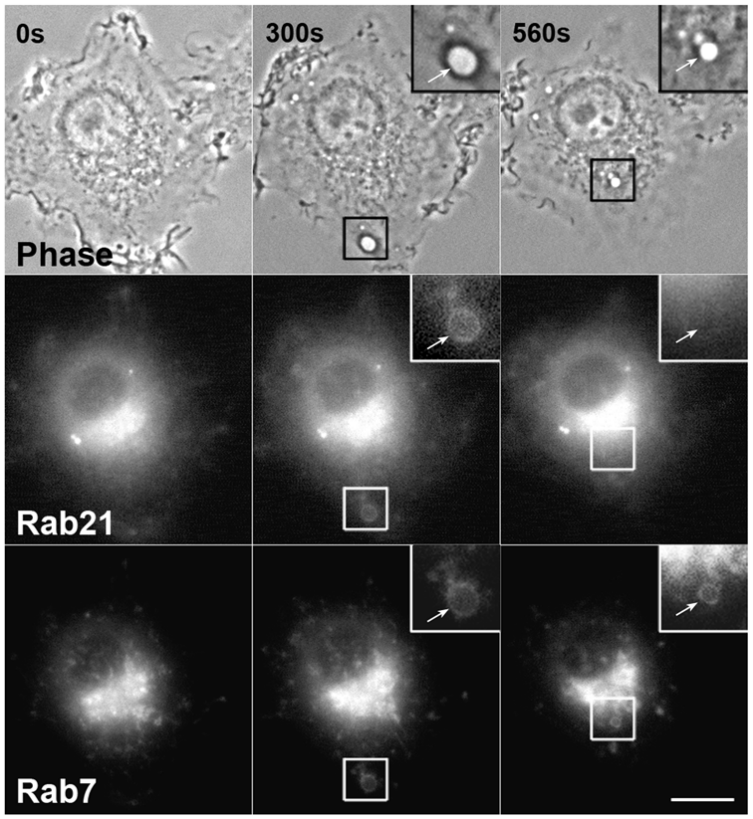
The association of Rab21 with macropinosomes is followed by Rab7 recruitment. RAW264 cells co-expressing CFP-Rab21 and YFP-Rab7 were observed by digital fluorescence microscopy. The association of Rab21 with macropinosomes was transient and followed by that of Rab7, which remained associated with the later stage of macropinosomes formation. The insets show higher-magnification images of the indicated regions of the cells. The arrows point to a macropinosome. Images are representative of three cells from three independent experiments. The corresponding movie is available (Movie S6). Scale bar: 10 µm.

It has been shown that macropinosomes sequentially acquire Rab5 and Rab7, a late endosomal marker, in EGF-stimulated HEK293 cells [6]. To reveal the distinct distributions of Rab21 and Rab7 in the macrophages during macropinocytosis, RAW264 cells were co-transfected with YFP-Rab7 and CFP-Rab21. The time-lapse images in Figure 6 show that, at the early stage of macropinosome maturation, high levels of Rab21 were present on the macropinosomes in which Rab7 was faintly seen. Although Rab7 was colocalized with Rab21 on some macropinosomes, the recruitment of Rab21 to the macropinosomes occurred earlier than that of Rab7 to some extent. Moreover, the reduction in Rab21 levels was accompanied by an increased level of Rab7 (Figure 6 and Movie S6). Taken together, these results indicate that the recruitment of Rab21 to macropinosomes is preceded by that of Rab5 but followed by that of Rab7.

### Rab21 is dissociated from the macropinosomes before the accumulation of Lamp1

**Figure 7:**
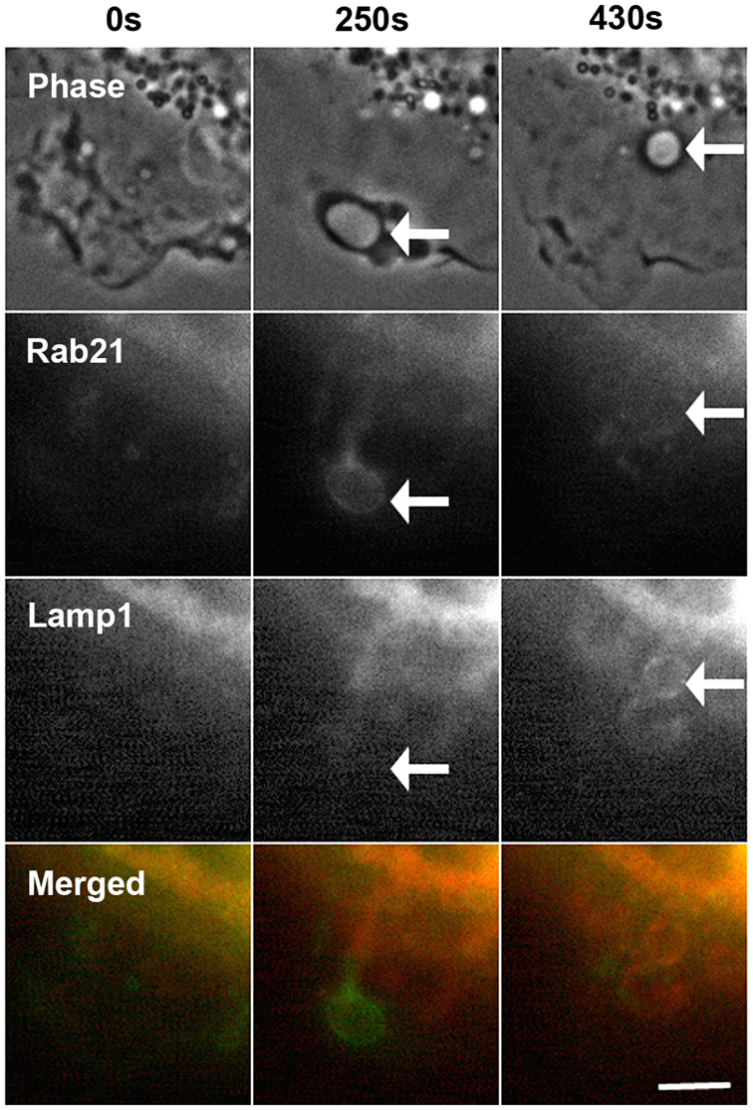
Rab21 is dissociated from the macropinosomes before Lamp1 accumulation. The dynamics of GFP-Rab21 (green) on the macropinosomes formed in RAW264 cells expressing CFP-Lamp1 (red) was examined by live-cell imaging. Shown is a representative series of images demonstrating the transient association of Rab21 with a macropinosome before the accumulation of Lamp1. Lamp1 remains associated with the membrane for extended periods of time. The arrows point to a macropinosome. Images are representative of three cells from three independent experiments. The corresponding movie is available (Movie S7). Scale bar: 5 µm.

Next, we compared the localization of CFP-lysosomal associated membrane protein 1 (Lamp1), a marker membrane protein for late endosomes and lysosomes, with GFP-Rab21 in co-transfected RAW264 cells. In RAW264 cells, Lamp1 is predominantly localized in the lysosomal membrane; however, Rab21 was found to be associated with newly formed macropinosomes which did not contain Lamp1 (Figure 7 and Movie S7). The fluorescence of GFP-Rab21 diminished on the macropinosomes when that of CFP-Lamp1 appeared on them. Also, no Lamp1-positive compartment was labeled with Rab21. These data suggest that the role of Rab21 in macropinocytosis is fulfilled at early and intermediate stages prior to the accumulation of Lamp1, and that Rab21 is dissociated from the macropinosomes when these acquire a lysosomal nature.

## Discussion

Both Rab5 and Rab21 are known to be early endosomal associates that regulate membrane trafficking in endocytic pathways. It is well established that Rab5 mainly localizes on early endosomes and regulates the homotypic fusion of early endosomes by interacting with EEA1 [32]–[34]. Furthermore, Rab5 is known to have several regulators and effectors such as Rabex-5, Rabaptin-5, Rabenosyn-5 and Rabankyrin-5 [22], [35], although little is known about those of Rab21. The present study revealed that Rab21 and Rab5 showed similar but not identical spatiotemporal dynamics in RAW264 macrophages during macropinocytosis. Recently, Porat-Shiliom et al. [36] have shown that Rab5 is recruited to macropinosomes after the loss of PI(4,5)P_2_ in HeLa cells over-expressing the active mutant of H-Ras (H-RasG12V) which induces membrane ruffling and macropinocytosis. In our study, it was found that Rab21 was largely colocalized with Rab5 on macropinosomes before the accumulation of Lamp1. Moreover, we observed that the loss of PI(4,5)P_2_ from the membrane was followed by the recruitment of Rab21 as well as Rab5. These facts imply that Rab21, like its close homologue Rab5, may play a role in regulating macropinocytosis at an early stage.

However, we observed that the onset of Rab21 recruitment to macropinosomes occurred slightly later than that of Rab5 in cells co-expressing CFP-Rab21 and GFP-Rab5. Intriguingly, while Rab5 recruitment was shown to occur before PI(3,4,5)P_3_ loss in cells expressing H-RasG12V [36], Rab21 was localized on macropinosomes after the loss of PI(3,4,5)P_3_ in RAW264 cells. Since the decrease in PI(3,4,5)P_3_ levels occurs shortly after macropinosome formation [13], [36], Rab21 could localize only on internalized macropinosomes. Therefore, it is unlikely that Rab21 is associated with circular ruffles and open macropinocytic cups. In good agreement with these facts, there was no significant alteration in the rate of macropinosome formation in the cells transfected with wild-type Rab21 or either of the mutants as compared to untransfected cells in our study. It has been reported that a dominant negative mutant of Rab5 inhibits the induction of circular ruffles known to be precursor forms of macropinosomes [37]. Rab5 is recruited to circular ruffles together with RN-tre, a Rab5 GTPase-activating protein (GAP) that mediates actin remodelling [12]. In addition, over-expression of Rabankyrin-5, a Rab5 effector, promotes macropinosome formation [22]. In contrast, over-expression of inactive Rab21 (GDP-bound mutant) did not inhibit circular ruffle or macropinosome formation in RAW264 cells in our study. Therefore, unlike Rab5, Rab21 seems not to be necessary for macropinosome formation. It is possible that Rab21 may play a unique role in macropinocytosis, although Rab21 shares some characteristics with Rab5.

**Figure 8:**
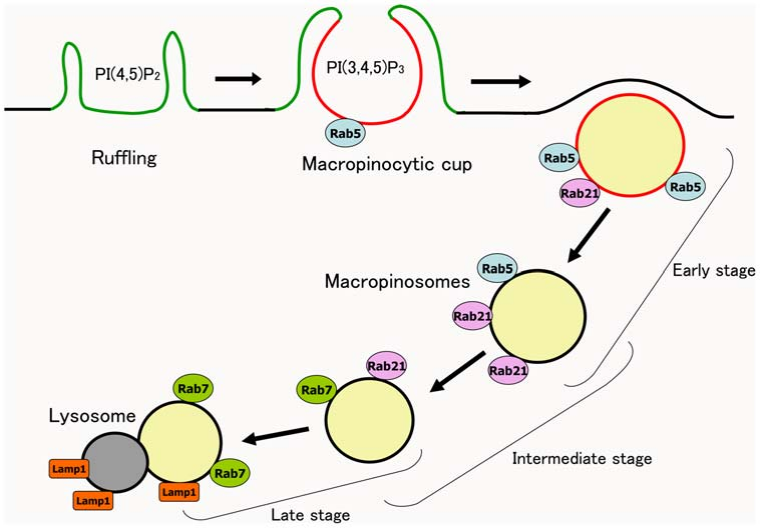
Schematic representation of the association of Rab proteins with macropinocytosis. Rab5 is localized on the inner membrane of macropinocytic cups and early macropinosomes. Rab7 is localized on later macropinosomes. Rab21 is localized on macropinosomes at an intermediate stage partially overlapping with Rab5 and Rab7, and then dissociates from the macropinosomes prior to Lamp1 acquisition by fusing with lysosomes. Green: PI(4,5)P_2_; red: PI(3,4,5)P_3_.

Another notable finding in our study is that Rab21 is recruited to the macropinosomes before the accumulation of Rab7 on the membrane. Then, Rab21 and Rab7 are colocalized on the macropinosomes at certain stages. Rab21 is dissociated from them before the acquisition of Lamp1, while Rab7 remains associated with the membrane even after the acquisition of Lamp1 [38]–[40]. Accordingly, it is likely that Rab5, Rab21 and Rab7 are sequentially associated with macropinocytosis to mediate the progressive maturation of the macropinosomes (Figure 8).

Although the mechanisms mediating Rab21 translocation to macropinosomes remain to be elucidated, important information can be inferred from the analysis of the localization of the Rab21 mutants, Rab21-T33N and Rab21-Q78L. The results presented here demonstrate that the GDP-bound mutant Rab21-T33N mainly localizes in the cytosol and the trans-Golgi network during macropinocytosis, whereas the GTP-bound mutant Rab21-Q78L is associated with macropinosomes, thereby providing evidence that GTP binding is required for Rab21 translocation, as has been found for other Rab proteins [18]. These results imply the involvement of guanine nucleotide exchange factors (GEFs), GTPase-activating proteins (GAPs) and/or some other interacting proteins in the regulation of Rab21 translocation. An intriguing finding in our study is that the association of Rab21 with macropinosomes is slightly preceded by that of Rab5 and persists even after Rab5 loss. Rab21 and Rab5 are phylogenetically close and have similar interaction partners. For instance, APPL1, which is an effector of Rab5 and present on newly formed macropinosomes, also interacts with Rab21 [41], [42]. Moreover, Rabex-5, characterized as a GEF for Rab5, has been shown to have equal GEF activity on Rab21 [43]. Recently, Zhang et al. have identified the VPS9-ankyrin-repeat protein (Varp) as a GEF for Rab21 [44]. They demonstrated that Varp preferentially interacts with the GDP-bound form of Rab21 and has a stronger guanine nucleotide exchange activity towards Rab21 than Rab5, leading to the activation and endosomal localization of Rab21. Such specificity and commonality in GEFs and effectors may be reflected in the slightly different temporal localizations between the two Rab proteins. In future studies, the definition of GEFs, GAPs and downstream effectors of Rab21 would be required for understanding the physiological consequences of Rab21 in the sequential activation of Rab proteins during macropinosome maturation.

In conclusion, our study demonstrated that Rab21 is transiently associated with macropinocytosis at an intermediate stage partially overlapping both the Rab5-acting stage and the Rab7-acting stage, and probably functions in macropinosome maturation rather than macropinosome formation. The macropinocytic pathway is coordinately regulated by several Rab GTPases, creating sequential Rab-effector links that mediate the progressive maturation of the organelles.

## Materials and Methods

### Reagents

Bovine serum albumin (BSA), rhodamine B isothiocyanate (RITC)-dextran (Mr ∼70,000) and Dulbecco's modified Eagle's medium (DMEM) were obtained from Sigma Chemical (St Louis, MO). Mouse recombinant macrophage colony stimulating factor (M-CSF) was obtained from R&D systems (Minneapolis, MN). All other reagents were purchased from Wako Pure Chemicals (Osaka, Japan) or Nacalai Tesque (Kyoto, Japan), unless otherwise indicated.

### Cell culture and drug treatments

Mouse macrophage RAW264 cells were cultured in DMEM supplemented with 10% heat-inactivated fetal bovine serum (FBS), 100 U/ml penicillin and 100 µg/ml streptomycin, as described in the manuals of the cell line bank. Before the experiments, the cells were serum-starved for 2 h in Ringer's buffer (RB) consisting of 155 mM NaCl, 5 mM KCl, 1 mM MgCl_2_, 2 mM Na_2_HPO_4_, 10 mM glucose, 10 mM HEPES pH 7.2 and 0.5 mg/ml BSA. Macropinocytosis was enhanced by adding 2000 U/ml M-CSF to the serum-starved RAW264 cells. To label the macropinosomes, RITC-dextran was added to RB at the concentration of 1 mg/ml for 10 min.

### DNA constructs and transfection

pEGFP-Rab21 (wild-type), pEGFP-Rab21-T33N (GDP-bound mutant) and pEGFP-Rab21-Q78L (GTP-bound mutant) were kindly provided by Dr. Arwyn T. Jones. pECFP-Rab21 was generated by the replacement of GFP with cyan fluorescent protein (CFP). pEYFP-Rab5, pEYFP-Rab7, pEYFP-Lamp1, pECFP-phospholipase Cδ1 (PLC) pleckstrin homology domain (PH) and pECFP-Akt-PH were generous gifts from Dr. Joel A. Swanson. pECFP-Lamp1 was generated by the replacement of yellow fluorescent protein (YFP) with CFP. The cDNA fragment comprising the entire coding region for rat Rab1B was generated by PCR amplification of rat cDNAs. The primers used were TCTCGAGCTATGAACCCCGAATATGACTAC and TGGATCCCTAGCAGCAGCCACCACTAG. The fragment was cloned into the XhoI and BamHI restriction sites of the pEGFP-C1 vector. All constructs were verified by sequencing. The RAW264 cells were transfected by electroporation. The cells were suspended at 5×10^6^ cells/ml in growth medium. Four hundred µl of the cell suspension were mixed with 5 µg of plasmid DNA in a 4-mm-gap electroporation cuvette. Electroporation was performed using an ECM 630 Electroporation System (BTX Harvard Apparatus, Inc., MA) at 300 V, 1000 µf and 25 Ω, to yield a time constant of ∼12 ms. Cells were then seeded onto 25-mm coverslips and maintained in the growth medium. Experiments were performed 24–48 h after transfection.

### Live-cell imaging

RAW264 cells were cultured on 25-mm circular coverslips and assembled in an RB-filled chamber on the thermo-controlled stage (Tokai Hit INU-ONI, Shizuoka, Japan) of an inverted epifluorescence microscope (Nikon TE300). Phase-contrast and fluorescence images of live cells were sequentially taken through a digital cooled CCD camera (Retiga Exi, QImaging, Surrey, BC, Canada) using shutters and filter wheels controlled by the MetaMorph imaging system (Molecular Devices, Downingtown, PA). In the cells co-expressing GFP- and CFP-fusion proteins, we confirmed that neither fluorescence signal was detected through the other filter set. Time-lapse images of phase-contrast and fluorescence microscopy were taken at 10 s intervals and assembled into QuickTime movies by the MetaMorph imaging system, as previously described [13]. At least 3 examples were observed in each experiment, and one representative video is shown.

## Supporting Information

Movie S1 Time-lapse movie showing the localization of GFP-Rab21 in RAW264 cells. Note the association of Rab21 with macropinosomes in the M-CSF-stimulated RAW264 cell. The images were collected at 10-s intervals. This is a representative of five cells, and similar results were obtained from three-independent experiments.
(2.55 MB MOV)

Movie S2 The association of GFP-Rab21 with macropinosomes in RAW264 cells without M-CSF stimulation. Rab21 was transiently associated with macropinosomes in unstimulated cells, although the frequency of macropinosome formation was lower than that in M-CSF-stimulated cells. This movie is a representative of three cells from two independent experiments.
(4.79 MB MOV)

Movie S3 Time-lapse movie showing the dynamics of Rab21 and PI(4,5)P2 during macropinocytosis. RAW264 cells co-expressing GFP-Rab21 (green) and CFP-PLC-PH (red) were stimulated with M-CSF. The GFP and CFP images were collected at 10-s intervals. This movie corresponds to Figure 4A, that is a representative of three independent experiments.
(1.47 MB MOV)

Movie S4 Time-lapse movie showing the dynamics of Rab21 and PI(3,4,5)P3 during macropinocytosis. RAW264 cells co-expressing GFP-Rab21 (green) and CFP-Akt-PH (red), which shows the localization of PI(3,4,5)P3, were stimulated with M-CSF. The GFP and CFP images were acquired every 10 s. This movie corresponds to Figure 4B, that is a representative of four independent experiments.
(2.99 MB MOV)

Movie S5 Time-lapse movie showing the dynamics of Rab21 and Rab5 during macropinocytosis. RAW264 cells co-expressing CFP-Rab21 (red) and YFP-Rab5 (green) were stimulated with M-CSF. The CFP and YFP images were acquired every 10 s. This movie corresponds to Figure 5, that is a representative of three independent experiments.
(2.01 MB MOV)

Movie S6 Time-lapse movie showing the dynamics of Rab21 and Rab7 during macropinocytosis. RAW264 cells co-expressing CFP-Rab21 (red) and YFP-Rab7 (green) were stimulated with M-CSF. The CFP and YFP images were acquired every 10 s. This movie corresponds to Figure 6, that is a representative of three independent experiments.
(3.23 MB MOV)

Movie S7 Time-lapse movie showing the dynamics of Rab21 and Lamp1 during macropinocytosis. The time-lapse images of RAW264 cells expressing GFP-Rab21 (green) and CFP-Lamp1 (red) were taken every 10 s after M-CSF stimulation. Rab21 is transiently associated with macropinosomes before the accumulation of Lamp1. This movie corresponds to Figure 7, that is a representative of three independent experiments.
(3.79 MB MOV)
